# Effect of Neutrophil-to-Lymphocyte Ratio on Post-TAVR Mortality and Periprocedural Pulmonary Hypertension

**DOI:** 10.1155/2024/4512655

**Published:** 2024-02-20

**Authors:** Xin Gao, Xiaoxiao Jiang, Zonglei Wu, Na Chen, Minghui Gong, Xu Zhao, Yan Liu, Ran Guo

**Affiliations:** ^1^Department of Cardiology, The First Affiliated Hospital of Dalian Medical University, Dalian, China; ^2^Department of Cardiovascular Surgery, The First Affiliated Hospital of Dalian Medical University, Dalian, China; ^3^Department of Cardiovascular Ultrasound, The First Affiliated Hospital of Dalian Medical University, Dalian, China

## Abstract

**Aims:**

To evaluate the impact of neutrophil-to-lymphocyte ratio (NLR) on periprocedural pulmonary hypertension (PH) and 3-month all-cause mortality in patients with aortic stenosis (AS) who underwent transcatheter aortic valve replacement (TAVR) and to develop a nomogram for predicting the mortality for these patients.

**Methods and Results:**

124 patients undergoing TAVR were categorized into three groups according to systolic pulmonary artery pressure (sPAP): Group I (no PH, *n* = 61) consisted of patients with no pre- and post-TAVR PH; Group II (improved PH, *n* = 35) consisted of patients with post-TAVR systolic pulmonary artery pressure (sPAP) decreased by more than 10 mmHg compared to pre-TAVR levels; and Group III (persistent PH, *n* = 28) consisted of patients with post-TAVR sPAP no decrease or less than 10 mmHg, or new-onset PH after the TAVR procedure. The risk of all-cause mortality within 3 months tended to be higher in Group II (11.4%) and Group III (14.3%) compared to Group I (3.3%) (*P*=0.057). The multinomial logistic regression analysis demonstrated a positive correlation between NLR and both improved PH (OR: 1.182, 95% CI: 1.036–1.350, *P*=0.013) and persistent PH (OR: 1.181, 95% CI: 1.032–1.352, *P*=0.016). Kaplan–Meier analysis revealed a significant association between higher NLR and increased 3-month all-cause mortality (16.1% vs. 3.1% in lower NLR group, *P*=0.021). The multivariable Cox regression analysis confirmed that NLR was an independent predictor for all-cause mortality within 3 months, even after adjusting for clinical confounders. A nomogram incorporating five factors (BNP, heart rate, serum total bilirubin, NLR, and comorbidity with coronary heart disease) was developed. ROC analysis was performed to discriminate the ability of the nomogram, and the AUC was 0.926 (95% CI: 0.850–1.000, *P* < 0.001).

**Conclusions:**

Patients with higher baseline NLR were found to be at an increased risk of periprocedural PH and all-cause mortality within 3 months after TAVR.

## 1. Introduction

The transcatheter aortic valve replacement (TAVR) has emerged as the preferred treatment option for patients of severe aortic stenosis (AS) who are at high surgical risk. Over time, the indication for TAVR has expanded to intermediate and low-risk patients as well [1–3]. The outcome of TAVR can be affected by various risk factors, and risk assessment plays a crucial role in clinical decision making. However, accurately assessing these risks remains a significant challenge before TAVR procedure.

Pulmonary hypertension (PH) occurs in approximately 50% of patients with left-sided heart disease and is commonly observed in patients with severe AS [4]. PH is already recognized as a predictor of adverse outcomes after TAVR [5–8]. PH due to the elevation of left ventricular (LV) end-diastolic pressure can be reduced following the TAVR procedure. Several recent studies have revealed that preexisting PH had prognostic impact on TAVR, affecting outcomes such as mortality and heart failure hospitalization [4]. Furthermore, persistent PH after TAVR has been demonstrated to have a stronger predictive value than baseline PH in patients undergoing TAVR procedure [9, 10]. It has been demonstrated that periprocedural PH was dynamic, and the periprocedural PH status significantly predicted the outcome of TAVR [11].

Evaluating peri-TAVR pulmonary artery pressure is of crucial significance in clinical practice. Even though invasive right heart catheterization is considered as the gold standard for diagnosing PH, the measurement of peak tricuspid regurgitation (TR) velocity using Doppler echocardiography, which is easier and noninvasive, remains the most commonly used method.

Inflammation has been linked to the development and prognosis of cardiovascular diseases [12, 13]. Previous studies have demonstrated a correlation between systemic inflammatory response syndrome after TAVR and adverse outcomes [14, 15]. However, the impacts of the preprocedural inflammatory state on periprocedural PH and TAVR outcomes have not been extensively studied. The neutrophil-to-lymphocyte ratio (NLR) has emerged as an easily obtainable and cost-effective biomarker derived from routine blood tests. It reflects a low-grade state of inflammation and has been reported to be associated with the prognosis of cardiovascular diseases [16, 17]. Therefore, in the current study, we aimed to evaluate the impact of baseline NLR on periprocedural PH and 3-month mortality in patients undergoing TAVR. Furthermore, we sought to develop a nomogram that could predict 3-month all-cause mortality in these patients.

## 2. Methods

### 2.1. Study Population and Data Collection

We retrospectively analyzed the medical records of 128 patients with degenerative aortic diseases, including AS and aortic regurgitation (AR), undergoing TAVR between January 2018 and December 2022, from the Cardiac Department of the First Affiliated Hospital of Dalian Medical University, and recorded the 3-month all-cause mortality. Two patients experienced TAVR procedure failure, and two patients died during the procedure. Therefore, a total of 124 patients were included in the final study. All 124 patients underwent echocardiography within 1 week before and after TAVR procedure. Selection criteria included patients with degenerative aortic diseases, specifically those with symptomatic severe AS characterized by an aortic valve area <0.8 cm^2^ and peak jet velocity >4 m/s or resting mean gradient >40 mm·Hg as determined by echocardiography. Additionally, patients with any degree of AS and severe AR were also included in this study. This study complies with the Declaration of Helsinki and was approved by the Ethics Committee of the First Affiliated Hospital of Dalian Medical University. Written informed consent was obtained from all patients.

### 2.2. TAVR Procedure

All patients, who had symptomatic AS with or without AR, had high surgical risk. The decision to undergo TAVR was made by a multidisciplinary heart team comprising of cardiologists, cardiovascular surgeons, echocardiographic and cardiac CT specialists, and anesthesiologists. The devices were delivered by the transfemoral approach. The implanted valves were VenusA-Valve (Venus Medtech Inc, Hangzhou, China) in the majority of the patients. The valve size was chosen based on a comprehensive assessment using cardiac CT and echocardiography.

### 2.3. Study Endpoint

The outcome of this study was 3-month all-cause mortality. Follow-up began on the day of the TAVR procedure, and information was obtained retrospectively from patients' medical records, clinic follow-up visits, or telephone interviews.

### 2.4. Biochemical Tests

Venous blood samples were collected from all patients after a fasting period of more than 8 hours and prior to the TAVR procedure. The blood samples were sent to the Laboratory Center at the First Affiliated Hospital of Dalian Medical University for analysis of various parameters, including blood routine items, renal function, hepatic function, blood lipids, uric acid (UA), B-type natriuretic peptide (BNP), and high-sensitivity troponin C (hs-cTnI). The NLR and platelet-lymphocyte-ratio (PLR) were calculated based on the obtained blood routine values. Renal function was assessed using the estimated glomerular filtration rate (eGFR), which was calculated using the following formula: eGFR (ml/min/1.73 m^2^) = 186 × (Scr/88.402)^−1.154^ × age^−0.203^ (×0.742 for females), where Scr represents the serum creatinine level.

### 2.5. Transthoracic Echocardiographic Assessment

Pre- and postprocedural echocardiographies were performed using a Vivid E9 ultrasound system (GE Vingmed Ultrasound, Horten, Norway). All echocardiographic measurements were performed following the guideline of the American Society of Echocardiography [18]. The maximum left atrial volume (LAV) was measured using the biplane modified Simpson's rule, and the LAV index (LAVI) was calculated by dividing the LAV by the body surface area (BSA). On the LV parasternal long axis view, the right ventricular (RV) diastolic diameter (RVD), LV diastolic diameter (LVD), septum thickness (IVST), and LV posterior wall thickness (LVPWT) were recorded. The following formula was used to calculate the LV mass (LVM): LVM = 0.8 × 1.04 [(LVD + IVST + LVPWT)^3^ − LVD^3^] + 0.6, and the LVMI (g/m^2^) was obtained as LVM/BSA. Main pulmonary artery diameter (PAD) was measured on the parasternal short axis view. The early diastolic transmitral flow velocity (*E*) was measured by pulsed-wave flow Doppler, the lateral mitral myocardial early diastolic velocity (*e*′) was obtained by pulsed-wave tissue Doppler at the apical four-chamber view, and the *E*/*e*′ ratio was then calculated. TR velocity was evaluated to diagnose PH, and the cutoff value for TR was determined to be 2.8 m/s, which corresponds to a systolic pulmonary artery pressure (sPAP) of 36 mmHg. A TR velocity exceeding 2.8 m/s, a defined threshold for diagnosing PH in accordance with American and European guidelines [8, 19], correlates with a sPAP of 36 mmHg. Furthermore, this established cutoff value has also been adopted and verified in previous studies [11, 20].

### 2.6. Statistical Analysis

The normality of the data was assessed using the Kolmogorov–Smirnov test. Continuous variables were presented as either mean ± standard deviation (SD) or the median and interquartile range (IQR; 25^th^–75^th^ percentile), according to the distribution of the variable. One-way ANOVA was used to evaluate the differences among normally distributed continuous variables, and post hoc pairwise comparison was carried out using the least significant difference (LSD) test. For nonnormally distributed variables, Kruskal–Wallis one-way ANOVA was used. The chi-square test was use to analyze categorical variables. A *P* value of less than 0.05 was considered as statistical significance.

Multinomial logistic regression was performed to identify the “Improved PH” and “Persistent PH” groups in comparison to the “No PH” group and to determine the predictors of the periprocedural PHT variation after TAVR. Kaplan–Meier curves were generated to illustrate the association between higher and lower NLR groups (according to the median of NLR) and 3-month all-cause mortality. Univariable Cox regression and multivariable analysis were performed to evaluate the impact of NLR on 3-month all-cause mortality. Three models were developed. Model 1 was adjusted for age, sex, and body mass index (BMI). Model 2 included Model 1 variables plus the history of hypertension, diabetes mellitus (DM), coronary heart disease (CHD), and atrial fibrillation (AF), BNP, and eGFR. Model 3 included Model 2 variables plus LVEF, LAVI, LVMI, *E*/*e*′, and trans-AV velocity. A multivariable Cox regression analysis was performed to create a nomogram for predicting 3-month all-cause mortality using predictors selected by Lasso regression. Furthermore, the receiver operating characteristic (ROC) curve was developed to evaluate the accuracy of the prediction. SPSS software 21.0 (SPSS, Chicago, Illinois, USA) and R (version 4.2.2, R Foundation for Statistical Computing, Vienna, Austria) were used to conduct the statistical analysis.

## 3. Results

### 3.1. Baseline Characteristics

After excluding 4 patients (2 patients due to death during the procedure, and 2 patients due to procedure failure), the remaining 124 patients were classified into three groups based on the alteration of sPAP pre- and post-TAVR procedure: Group I (no PH, *n* = 61) consisted of patients with no pre- and post-TAVR PH; Group II (improved PH, *n* = 35) consisted of patients with post-TAVR sPAP decreased by more than 10 mmHg compared to their pre-TAVR levels; and Group III (persistent PH, *n* = 28) consisted of patients with post-TAVR sPAP no decrease or less than 10 mmHg, or new-onset PH after the TAVR procedure. In this study, 122 patients exhibited severe AS, while the remaining 2 patients presented with mild AS with concomitant severe AR and morphological suitable for TAVR. Within Group III, 8 patients developed new-onset PH. These individuals displayed higher BMI (25.02 ± 3.57 kg/m^2^) and SBP (141 ± 21 mmHg), increased NLR (8.44 ± 10.09) and PLR (93.47 ± 81.75), elevated LAVI (28.22 ± 10.97 ml/m^2^) and LVEF (54.63 ± 8.28%), along with reduced eGFR (58.74 ± 36.55) (ml/min/1.73 m^2^), and relatively lower LVMI (130.43 ± 36.00 g/m^2^). However, statistical analysis between subgroups was not conducted due to the limited number of patients.

Table 1 summarizes the baseline characteristics, biochemical tests, and echocardiographic parameters of the patients. The three groups were comparable in terms of age, gender, BMI, and medical history, including hypertension, DM, CHD, and AF. Clinical characteristics such as systolic blood pressure (SBP), diastolic blood pressure (DBP), and heat rate (HR) were also similar among the three groups. However, routine blood biochemistry test demonstrated significant differences in lymphocyte count (*P*=0.008), NLR (*P*=0.024), PLR (*P*=0.017), the red cell distribution width_Standard Deviation (RDW_SD) (*P*=0.001), the red cell distribution width_Coefficient of Variation (RDW_CV) (*P*=0.001), aspartate aminotransferase (AST) (*P*=0.008), serum total bilirubin (T-BIL) (*P*=0.028), BNP (*P* < 0.001), and UA (*P*=0.025) among three groups.

The baseline echocardiographic data show no significant differences in RVD, LVMI, trans-AV velocity, peak/average AV pressure, and right ventricular systolic pressure (RVSP) among the three groups. LAVI (*P* < 0.001) and *E*/*e*′ ratio (*P*=0.004) were significantly increased in Group II and Group III compared to Group I; however, the differences between Group II and III were not significant. LVEF was significantly lower in Group II than in Group I and Group III (*P*=0.002). PAD was significantly increased in Group III than in Group I (*P*=0.024).

At 3 months, 10 patients (8.1%) had died, with causes including 2 cases of acute heart failure, 3 cases of acute myocardial infarction, 2 instances of sudden cardiac death, and 3 cases with an unknown cause. In addition, 15 patients (12.1%) required pacemaker implantation, 27 patients (21.8%) experienced new-onset complete left bundle branch block (CLBBB), and 3 patients (2.4%) developed new-onset atrial fibrillation. None of patients had symptomatic stroke.

### 3.2. The Prediction of PH

Univariable and multivariable logistic regression analyses were conducted to identify factors associated with pre- and post-TAVR PH. As shown in Table 2, BMI, NLR, PLR, r-glutamyltranspeptidase (r-GT), LAVI, PAD, and peak AV pressure were independently associated with pre-TAVR PH. Moreover, RDW_SD and PAD were independently associated with post-TAVR PH.

Univariable and multivariable multinomial logistic regression analyses were performed to determine the predictors for periprocedural PH variation. As illustrated in Table 3, patients with higher NLR had increased odds of developing PH, including both improved PH (OR: 1.182, 95% CI: 1.036–1.350, *P*=0.013) and persistent PH (OR: 1.181, 95% CI: 1.032–1.352, *P*=0.016), compared to those without PH. Additionally, patients with increased peak AV velocity were also more likely to develop PH. Higher PAD was associated with increased odds of developing persistent PH, while higher PLR was associated with increased odds of developing improved PH compared to those without PH.

### 3.3. The Impact of NLR on 3-Month All-Cause Mortality

Table 4 demonstrates the 3-month all-cause mortality for different PH groups. The 3-month all-cause mortality tended to be higher in PH group, which included pre- and post-TAVR, and periprocedural PH groups. However, the differences were not statistically significant, possibly due to the limited number of patients in the study.

**Figure 1:**
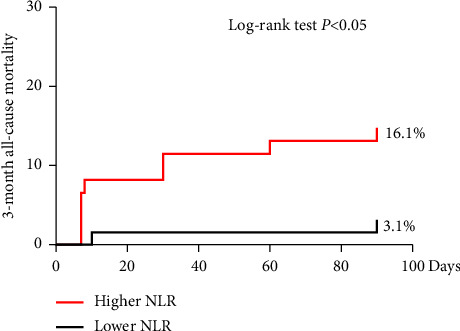
Kaplan–Meier curve of 3-month all-cause mortality. NLR, neutrophil-to-lymphocyte ratio.

Three month follow-up was completed for all 124 patients (100%). Kaplan–Meier curve of 3-month all-cause mortality is shown in Figure 1. Higher NLR was significantly associated with increased mortality (16.1% vs. 3.1% in lower NLR group, *P*=0.021). We used univariate and multivariable cox regression to evaluate the impact of NLR on 3-month all-cause mortality. The results indicated that increased NLR was identified as an independent predictor of mortality, even after adjusting for various clinical founders, including age, gender, BMI, medical history, BNP, eGFR, LVEF, LAVI, LVMI, *E*/*e*′ ratio, and peak AV velocity. The hazard ratio (HR) for 3-month all-cause mortality in relation to NLR was 1.142 (95% CI, 1.056–1.235) with a statistical significance (*P*=0.001) (Table 5).

### 3.4. Nomogram for Predicting 3-Month All-Cause Mortality

**Figure 2:**
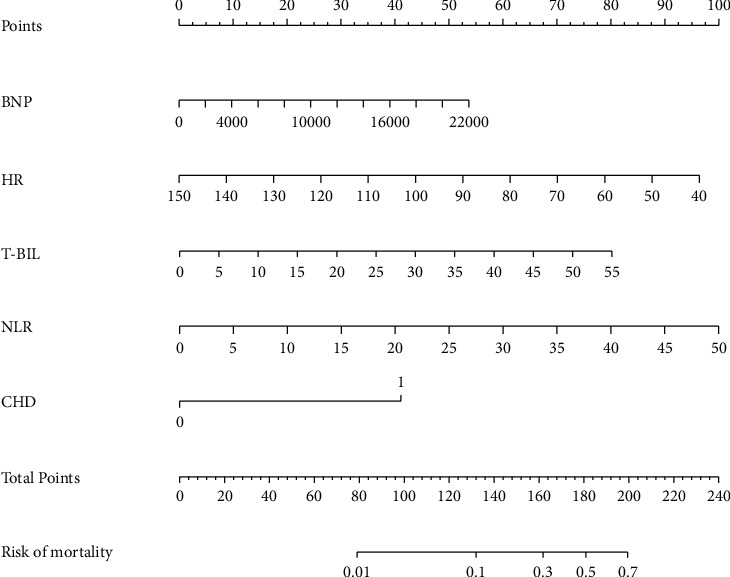
Nomogram for predicting 3-month all-cause mortality after TAVR. BNP, B-type natriuretic peptide; HR, heart rate; T-BIL, serum total bilirubin; NLR, neutrophil-to-lymphocyte ratio; CHD, coronary heart disease; TAVR, transcatheter aortic valve replacement.

**Figure 3:**
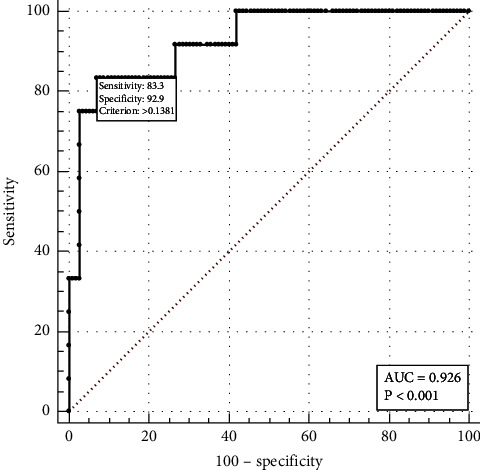
ROC for discriminating the predictive ability of the nomogram.

Lasso regression was performed to select factors from 45 potential variables based on clinical significance, and a nomogram incorporating five factors (BNP, HR, T-BIL, NLR, and CHD) was developed (Figure 2). ROC analysis was performed to discriminate the predictive ability of the nomogram, and the area under the ROC (AUC) was 0.926 (95% CI: 0.850–1.000, *P* < 0.001) (Figure 3).

## 4. Discussion

This retrospective study was conducted at a single center. Our findings indicated that baseline NLR before TAVR procedure was associated with pre-TAVR PH, periprocedural PH variation, and 3-month all-cause mortality. Additionally, we developed a nomogram for predicting the 3-month all-cause mortality in patients undergoing TAVR based on routine clinical examinations. This nomogram incorporated five factors (BNP, HR, T-BIL, NLR, and CHD) and demonstrated a reliable predictive value.

In patients with AS, PH is not uncommon. AS causes an increase in LV pressure, which subsequently transmits high pressure to the pulmonary circulation by elevating LA pressure and reducing LA compliance, elevates pulmonary arteriolar tone, and then induces the reactive PH. This belongs to Group 2 PH, which is associated with left heart disease [8]. AS initially leads to the development of isolated postcapillary PH (IpcPH), and over time the persistent LA changes contribute to functional and structural remodeling of the entire pulmonary circulation and an increase in pulmonary vascular resistance. Ultimately, this can result in the development of combined post- and precapillary PH (CpcPH). During the peri-TAVR procedure, PH is a dynamic condition. TAVR is effective in alleviating aortic stenosis and improving hemodynamics by reducing the overload of the left ventricle, left atrium, and pulmonary vasculature. After the TAVR procedure, it is expected that pulmonary pressure will decrease. However, despite the successful TAVR intervention, some patients still experience persistent PH or develop new-onset PH. In the current study, 43.5% AS patients (54 patients) had PH before TAVR, and 64.8% (35 patients) of these patients had sPAP improved after TAVR procedure. Additionally, 56.5% AS patients (70 patients) had no PH before TAVR; however, 12.9% (9 patients) of these patients developed PH (new-onset PH) after TAVR procedure. The failure of improvement in sPAP following TAVR may be attributed to the occurrence of CpcPH, indicating significant and irreversible remodeling of pulmonary vasculature.

The correlation of PH and adverse outcomes of TAVR has been extensively studied and proved. Pre-TAVR PH was reported to be related to the adverse outcomes [21], and post-TAVR PH had a more significantly prognostic prediction for clinical outcomes [11]. Failure to decrease PH after the TAVR procedure was also found to be correlated with increased mortality [11]. The plausibility of the PH recovery following TAVR was considered to be dependent on the severity of pulmonary circulation remodeling and left ventricular myocardial damage induced by AS [5, 22]. Ujihira et al.'s study [23] demonstrated that patients who experienced an increase in sPAP at 1 month after successful TAVR procedure were at a higher risk of mortality and rehospitalization within 1 year, regardless of their baseline sPAP. In the current study, the 3-month all-cause mortality increased progressively from no PH group (3.3%) to the improved PH group (11.4%), and then to the persistent PH group (14.3%). However, the differences showed no statistical significance, possibly due to the limited number of patients.

The present study also revealed that an inflammation biomarker, NLR, not only was related to periprocedural PH but also emerged as a significant predictor of 3-month all-cause mortality in patients following TAVR. Inflammation contributes vitally to age-related chronic disorders and cardiovascular diseases, including AS and PH [24, 25]. Inflammatory responses are not limited to PH related to immune system disorders (connective tissue disease-related PH). They can also occur in other forms of PH [26], including Group II PH, which is associated with left heart disease [27–30]. In PH, there is an elevation of circulating inflammatory factors, and inflammation is frequently associated with PH both in animal models and clinical settings [29, 30]. Furthermore, following TAVR, a systemic inflammatory response can also occur, which has been consistently and independently correlated with higher mortality in previous studies [14, 15]. NLR, a simple indicator of systemic inflammation, has been previously investigated as a predictor for organ damage and adverse outcomes in cardiovascular diseases [31–33]. Several studies have examined the relationship between NLR and outcomes following TAVR, consistently revealing that higher baseline NLR was independently correlated with adverse outcomes, including all-cause mortality and heart failure rehospitalization [34–36]. A potential pathophysiologic explanation involves: AS, which is a chronic disease marked by prolonged inflammation, can result in reduced physical capabilities, appetite loss, and a decline in nutritional status, particularly among older patients. The NLR, a marker of chronic low-grade inflammation, is also considered an indicator of the onset of geriatric frailty, contributing to elevated mortality.

Furthermore, in the current study, we developed a nomogram that effectively integrated multiple factors to construct a robust predictive model for 3-month all-cause mortality based on routine clinical examinations. Our findings revealed that patients with elevated baseline levels of NLR, BNP, T-BIL, HR, and comorbidity with CHD exhibited a higher risk of mortality. Therefore, the current study has significant implications in daily clinical practice and patient care in the context of TAVR. This study contributes to the identification of high-risk AS patients prior to TAVR based on easily obtainable routine tests. It not only contributes to the enhancement of high-risk patients' management and improvement of treatment outcomes but also is valuable for clinical decision making and care strategies.

## 5. Limitations

The current study has several potential limitations. Firstly, it was a single-center retrospective study that included a limited number of patients and had a short-term follow-up. Therefore, the conclusions drawn from this study need to be further investigated through large-scale clinical trials with an extended duration of patients' follow-up. Secondly, we utilized noninvasive echocardiographic methods to evaluate sPAP and diagnose PH, instead of employing the more accurate technique of right heart catheterization. Finally, the study only focused on the biomarkers obtained from routine blood tests and did not analyze other inflammatory biomarkers, such as C-reactive protein, erythrocyte sedimentation rate, and interleukin-6.

## 6. Conclusions

Patients with higher baseline NLR were found to be at an increased risk of periprocedural PH and all-cause mortality within 3 months.

The nomogram developed based on clinical routine assessment in this study is believed to contribute to the identification of high-risk AS patients before the TAVR procedure, and the easily obtainable predictive model may serve as an effective tool for clinical practice.

## Figures and Tables

**Table 1:** Clinical characteristics of patients.

	No PH (*n* = 61)	Improved PH (*n* = 35)	Persistent or new-onset PH (*n* = 28)	*P* value
Age (years)	78 ± 7	81 ± 7	79 ± 9	0.149
Male sex, *n* (%)	31 (50.0%)	17 (27.4%)	14 (22.6%)	0.892
BMI (kg/m^2^)	24.57 (22.57, 27.39)	22.10 (21.37, 24.63)	23.96 (20.17, 26.72)	0.068
Medical history				
HT, *n* (%)	41 (67.2%)	23 (62.2%)	17 (60.7%)	0.796
DM, *n* (%)	19 (31.1%)	11 (29.7%)	6 (21.4%)	0.630
CHD, *n* (%)	19 (31.1%)	13 (35.1%)	9 (32.1%)	0.919
AF, *n* (%)	7 (11.5%)	7 (18.9%)	7 (25.0%)	0.257
Clinical characteristics				
SBP (mmHg)	138 ± 23	133 ± 24	135 ± 22	0.547
DBP (mmHg)	75 ± 13	71 ± 15	73 ± 11	0.279
HR (bpm)	76 ± 16	83 ± 18	79 ± 17	0.104
FPG (mmol/L)	5.33 (4.67, 8.02)	5.59 (4.84, 7.36)	5.38 (5.00, 7.00)	0.916
WBC (×10^9^/L)	6.77 ± 0.33	6.26 ± 0.32	7.49 ± 0.55	0.150
RBC (×10^12^/L)	4.12 ± 0.08	4.01 ± 0.10	3.97 ± 0.15	0.504
HB (g/L)	125.62 ± 2.22	122.26 ± 3.13	117.46 ± 4.82	0.196
PLT (×10^9^/L)	191.45 ± 6.70	166.71 ± 8.77	199.43 ± 14.05	0.059
RDW_SD (fL)	43.40 (41.40, 45.85)	45.60 (43.10, 50.90)^*∗*^	47.35 (43.00, 52.40)^*∗*^	**0.001**
RDW_CV	0.13 (0.12, 0.14)	0.13 (0.13, 0.14)	0.14 (0.13, 0.16)^*∗∗*^	**0.001**
MPV (fL)	10.50 (9.90, 11.20)	10.80 (9.90, 11.80)	10.90 (10.23, 11.20)	0.274
PDW (fL)	12.43 ± 2.31	13.26 ± 2.65^*∗*^	12.70 ± 2.11^*∗*^	0.262
NEUT (×10^9^/L)	4.05 (3.21, 5.50)	4.32 (3.58, 5.69)	4.60 (3.79, 7.02)	0.295
LYM (×10^9^/L)	1.73 ± 0.91	1.25 ± 0.65	1.30 ± 0.75	**0.008**
NLR	2.74 (1.67, 4.29)	3.48 (2.54, 5.68)	5.78 (2.09, 8.89)	**0.024**
PLR	65.21 (43.58, 111.22)	46.56 (29.18, 63.39)^*∗*^	37.99 (18.41, 96.35)	**0.017**
ALT (U/L)	16.00 (11.00, 25.50)	23.00 (11.00, 40.50)	27.00 (17.00, 37.00)	0.181
AST (U/L)	19.00 (16.00, 25.25)	28.00 (19.00, 46.00)^*∗*^	27.00 (19.00, 37.00)	**0.008**
T-BIL (umol/L)	12.44 ± 1.01	16.87 ± 2.53^*∗*^	15.80 ± 1.41	**0.028**
r-GT (U/L)	21.00 (14.00, 34.75)	33.00 (18.80, 63.50)	31.50 (25.50, 40.00)	0.107
TP (g/L)	65.75 ± 1.85	64.71 ± 1.58	69.37 ± 2.55	0.246
ALB (g/L)	39.10 (37.20, 41.20)	39.00 (37.55, 40.40)	40.10 (37.28, 42.13)	0.370
TC (mmol/l)	4.71 ± 1.21	4.53 ± 1.21	4.70 ± 1.12	0.783
TG (mmol/l)	1.25 (0.88, 1.64)	0.95 (0.77, 1.29)	0.94 (0.76, 1.53)	0.065
HDL-C (mmol/l)	1.15 ± 0.36	1.08 ± 0.27	1.15 ± 0.28	0.560
LDL-C (mmol/l)	2.58 ± 0.83	2.55 ± 0.89	2.60 ± 0.83	0.981
UA (mmol/L)	340 ± 109	416 ± 204^*∗*^	416 ± 161^*∗*^	**0.025**
Scr (*μ*mol/l)	72 (64, 86)	73 (63, 129)	79 (63, 117)	0.436
eGFR (ml/min/1.73 m^2^)	84.56 ± 29.45	77.45 ± 36.32	71.01 ± 36.61	0.188
BNP (ng/L)	300 (131, 937)	1923 (523, 3164)^*∗∗*^	1015 (379, 2172)^*∗*^	**<0.001**
Hs-cTnI (*μ*g/L)	0.06 (0.19, 0.35)	0.13 (0.06, 0.39)	0.11 (0.02, 0.53)	0.247
Baseline echocardiographic data				
RVD (mm)	17 ± 2	18 ± 2	17 ± 2	0.078
LAVI (ml/m^2^)	31.45 ± 9.15	40.26 ± 10.56^*∗∗*^	39.74 ± 13.16^*∗∗*^	**<0.001**
LVMI (g/m^2^)	129 (112, 153)	149 (120, 180)	144 (114, 163)	0.350
LVEF (%)	58 (48, 58)	48 (37, 55)^*∗*^	55 (49, 58)^#^	**0.002**
*E*/*e*′ ratio	15 ± 6	20 ± 8^*∗*^	20 ± 7^*∗*^	**0.004**
AV velocity (m/s)	4.48 ± 0.84	4.75 ± 1.12	4.81 ± 0.82	0.191
Peak AV pressure (mmHg)	83.53 ± 28.09	95.54 ± 40.49	95.79 ± 30.58	0.130
Average AV pressure (mmHg)	50.33 ± 17.00	58.66 ± 26.18	58.04 ± 21.06	0.111
PAD (mm)	23 (21, 25)	24 (22, 26)	25 (23, 27)^*∗*^	**0.024**
RVSP (mm)	—	55 ± 14	51 ± 8	0.166

PH, pulmonary hypertension; BMI, body mass index; HT, hypertension; DM, diabetes mellitus; CHD, coronary heart disease; AF, atrial fibrillation; SBP, systolic blood pressure; DBP, diastolic blood pressure; HR heart rate; FPG, fast plasma glucose; WBC, white blood cell count; RBC, red blood cell count; HB, hemoglobin; PLT, platelet; RDW_SD, the red cell distribution width_Standard Deviation; RDW_CV, the red cell distribution width_Coefficient of Variation; MPV, mean platelet volume; PDW, platelet distribution width; NEUT, neutrophil count; LYM, lymphocyte count; NLR, neutrophil-to-lymphocyte ratio; PLR, platelet-lymphocyte-ratio; ALT, alanine aminotransferase; AST, aspartate aminotransferase; T-BIL, serum total bilirubin; r-GT, r-glutamyltranspeptidase; TP, total protein; ALB, albumin; TC, total cholesterol; TG, triglyceride; HDL-C, high-density lipoprotein cholesterol; LDL-C, low-density lipoprotein cholesterol; UA, uric acid; Scr, serum creatinine; eGFR, estimated glomerular filtration rate; BNP, B-type natriuretic peptide; hs-cTnI, high-sensitivity troponin C; RVD, right ventricular diastolic diameter; LAVI, left atrial volume index; LVMI, left ventricular mass index; LVEF, left ventricular ejection fraction; *E*/*e*′, early diastolic transmitral flow velocity/lateral mitral annular myocardial velocity; AV, aortic valve; PAD, pulmonary artery diameter; RVSP, right ventricular systolic pressure. ^*∗*^*P* < 0.05, vs. Group I; ^#^*P* < 0.05, vs. Group II. ^*∗∗*^*P* < 0.001, vs. Group I. The bold values indicate statistical significances (*P* < 0.05) among the three groups.

**Table 2:** The prediction of pre- and post-TAVR pulmonary hypertension.

Variables	Pre-TAVR PH			Post-TAVR PH		
	OR	95% CI	*P* value	OR	95% CI	*P* value
BMI (kg/m^2^)	0.747	0.585–0.952	**0.018** ^*∗*^	0.892	0.771–1.032	0.124
HB (g/L)				0.993	0.968–1.018	0.582
PLR	0.988	0.976–1.000	**0.045** ^*∗*^			
NLR	1.266	1.047–1.530	**0.015** ^*∗*^			
RDW_SD	1.073	0.970–1.186	0.172	1.083	1.000–1.173	**0.050** ^*∗*^
BNP (ng/L)	1.000	0.999–1.001	0.798			
TG (mmol/l)	0.660	0.233–1.867	0.433			
UA (mmol/L)	0.998	0.992–1.004	0.486	1.001	0.998–1.004	0.493
ALT	1.009	0.964–1.056	0.714			
T-BIL	1.038	0.920–1.171	0.543			
r-GT	1.031	1.008–1.053	**0.007** ^*∗*^			
RVD (mm)	1.142	0.749–1.742	0.537			
LAVI (ml/m^2^)	1.097	1.006–1.197	**0.036** ^*∗*^	1.000	0.953–1.049	0.992
PAD (mm)	1.574	1.096–2.261	**0.014** ^*∗*^	1.241	1.035–1.488	**0.020** ^*∗*^
LVEF (%)	0.953	0.873–1.040	0.280			
*E*/*e*′ ratio	1.083	0.971–1.208	0.154	1.050	0.978–1.127	0.175
Peak AV pressure (mmHg)	1.050	1.017–1.084	**0.003** ^*∗*^			

TAVR, transcatheter aortic valve replacement; PH, pulmonary hypertension; BMI, body mass index; HB, hemoglobin; NLR, neutrophil-to-lymphocyte ratio; PLR, platelet-lymphocyte-ratio; RDW_SD, the red cell distribution width_Standard Deviation; BNP, B-type natriuretic peptide; TG, triglyceride; UA, uric acid; ALT, alanine aminotransferase; T-BIL, serum total bilirubin; r-GT, r-glutamyltranspeptidase; RVD, right ventricular diastolic diameter; LAVI, left atrial volume index; PAD, pulmonary artery diameter; LVEF, left ventricular ejection fraction; *E*/*e*′, early diastolic transmitral flow velocity/lateral mitral annular myocardial velocity; AV, aortic valve. ^*∗*^*P* < 0.05, vs. No PH. The bold values indicate statistical significances (*P* < 0.05).

**Table 3:** The prediction of periprocedural pulmonary hypertension variation.

Variables	Improved PH			Persistent PH		
	OR	95% CI	*P* value	OR	95% CI	*P* value
BMI (kg/m^2^)	0.873	0.730–1.044	0.137	0.865	0.721–1.039	0.120
PLT	0.997	0.987–1.008	0.629	1.005	0.996–1.015	0.278
PLR	0.987	0.977–0.997	**0.010** ^*∗*^	0.994	0.985–1.003	0.208
NLR	1.182	1.036–1.350	**0.013** ^*∗*^	1.181	1.032–1.352	**0.016** ^*∗*^
RDW_SD	1.028	0.927–1.139	0.566	1.089	0.991–1.196	0.075
BNP (ng/L)	1.000	1.000–1.001	0.430	1.000	1.000–1.001	0.728
UA (mmol/L)	1.000	0.995–1.005	0.984	1.003	0.998–1.008	0.230
ALT	1.031	0.989–1.075	0.147	1.018	0.977–1.061	0.391
T-BIL	1.059	0.962–1.165	0.243	1.055	0.955–1.166	0.292
RVD (mm)	1.223	0.863–1.735	0.219	1.074	0.772–1.493	0.672
LAVI (ml/m^2^)	1.038	0.971–1.110	0.274	1.036	0.968–1.109	0.301
PAD (mm)	1.117	0.870–1.434	0.384	1.349	1.061–1.715	**0.015** ^*∗*^
LVEF (%)	1.005	0.942–1.072	0.881	1.069	0.996–1.148	0.065
*E*/*e*′ ratio	1.075	0.975–1.186	0.146	1.039	0.941–1.148	0.447
AV velocity (m/s)	2.282	1.168–4.456	**0.016** ^*∗*^	2.042	1.043–3.998	**0.037** ^*∗*^

No PH group as a referent. PH, pulmonary hypertension; BMI, body mass index; PLT, platelet; PLR, platelet-lymphocyte-ratio; NLR, neutrophil-to-lymphocyte ratio; RDW_SD, the red cell distribution width_Standard Deviation; BNP, B-type natriuretic peptide; UA, uric acid; ALT, alanine aminotransferase; T-BIL, serum total bilirubin; RVD, right ventricular diameter; LAVI, left atrial volume index; PAD, pulmonary artery diameter; LVEF, left ventricular ejection fraction; *E*/*e*′, early diastolic transmitral flow velocity/lateral mitral annular myocardial velocity; AV, aortic valve. ^*∗*^*P* < 0.05, vs. No PH. The bold values indicate statistical significances (*P* < 0.05).

**Table 4:** 3-month all-cause mortality of different PH groups.

	Pre-TAVR PH groups			Post-TAVR PH groups			Peri-TAVR PH variation groups			
	No PH (*n* = 70)	PH (*n* = 56)	*P*	No PH Z(*n* = 90)	PH (*n* = 34)	*P*	No PH (*n* = 61)	Improved PH (*n* = 35)	Persistent PH (*n* = 28)	*P*
3-month all-cause mortality	4 (5.7%)	8 (14.3%)	0.131	6 (6.7%)	4 (11.8%)	0.460	2 (3.3%)	4 (11.4%)	4 (14.3%)	0.057

TAVR, transcatheter aortic valve replacement; PH, pulmonary hypertension.

**Table 5:** Predictive values of NLR for 3-month all-cause mortality.

	Univariable Cox regression model		Multivariable Cox regression model 1		Multivariable Cox regression model 2		Multivariable Cox regression model 3	
	HR (95% CI)	*P* value	HR (95% CI)	*P* value	HR (95% CI)	*P* value	HR (95% CI)	*P* value
NLR	1.097 (1.058–1.138)	<0.001	1.091 (1.048–1.137)	<0.001	1.113 (1.050–1.180)	<0.001	1.142 (1.056–1.235)	0.001

Model 1, adjustment for age, sex, and BMI. Model 2, adjustment for Model 1 plus HT, DM, CHD, AF, BNP, and eGFR. Model 3, adjustment for Model 2 plus LAVI, LVEF, LVMI, *E*/*e*′, and peak AV velocity. NLR, neutrophil-to-lymphocyte ratio; BMI, body mass index; HT, hypertension; DM, diabetes mellitus; CHD, coronary heart disease; AF, atrial fibrillation; BNP, B-type natriuretic peptide; eGFR, estimated glomerular filtration rate; LAVI, left atrial volume index; LVEF, left ventricular ejection fraction; LVMI, left ventricular mass index; *E*/*e*′, early diastolic transmitral flow velocity/lateral mitral annular myocardial velocity; AV, aortic valve.

## Data Availability

The data used to support the findings of this study are available on request from the corresponding authors.

## References

[1] Popma J. J., Deeb G. M., Yakubov S. J. (2019). Transcatheter aortic-valve replacement with a self-expanding valve in low-risk patients. *New England Journal of Medicine*.

[2] Mack M. J., Leon M. B., Thourani V. H. (2019). Transcatheter aortic-valve replacement with a balloon-expandable valve in low-risk patients. *New England Journal of Medicine*.

[3] Leon M. B., Smith C. R., Mack M. J. (2016). Transcatheter or surgical aortic-valve replacement in intermediate-risk patients. *New England Journal of Medicine*.

[4] Dahiya G., Kyvernitakis A., Elhamdani A. (2023). Prognostic role of pulmonary hemodynamics before transcatheter aortic valve replacement among patients with severe aortic stenosis. *The Journal of Heart and Lung Transplantation*.

[5] O’Sullivan C. J., Wenaweser P., Ceylan O. (2015). Effect of pulmonary hypertension hemodynamic presentation on clinical outcomes in patients with severe symptomatic aortic valve stenosis undergoing transcatheter aortic valve implantation: insights from the new proposed pulmonary hypertension classification. *Circulation: Cardiovascular Interventions*.

[6] Abdelkarim I., Althouse A. D., Thoma F. W. (2017). The importance of invasive hemodynamics for pulmonary hypertension screening in TAVR patients. *Journal of the American College of Cardiology*.

[7] Levy F., Bohbot Y., Sanhadji K. (2018). Impact of pulmonary hypertension on long-term outcome in patients with severe aortic stenosis. *European Heart Journal Cardiovascular Imaging*.

[8] Humbert M., Kovacs G., Hoeper M. M. (2022). 2022 ESC/ERS Guidelines for the diagnosis and treatment of pulmonary hypertension. *European Heart Journal*.

[9] Alushi B., Beckhoff F., Leistner D. (2019). Pulmonary hypertension in patients With Severe aortic stenosis: prognostic Impact after Transcatheter Aortic valve replacement. *Journal of the American College of Cardiology: Cardiovascular Imaging*.

[10] Masri A., Abdelkarim I., Sharbaugh M. S. (2018). Outcomes of persistent pulmonary hypertension following transcatheter aortic valve replacement. *Heart*.

[11] Miyamoto J., Ohno Y., Kamioka N. (2022). Impact of periprocedural pulmonary hypertension on outcomes after transcatheter aortic valve replacement. *Journal of the American College of Cardiology*.

[12] Murphy S. P., Kakkar R., McCarthy C. P., Januzzi J. L. (2020). Inflammation in heart failure: JACC state-of-the-art review. *Journal of the American College of Cardiology*.

[13] Sorysz D., Januszek R., Sowa-Staszczak A. (2021). The usefulness of [(18)F]F-fluorodeoxyglucose and [(18)F]F-sodium fluoride positron emission tomography imaging in the assessment of early-stage aortic valve degeneration after transcatheter aortic valve implantation (TAVI)-Protocol description and preliminary results. *Journal of Clinical Medicine*.

[14] Lindman B. R., Goldstein J. S., Nassif M. E. (2015). Systemic inflammatory response syndrome after transcatheter or surgical aortic valve replacement. *Heart*.

[15] Sinning J. M., Scheer A. C., Adenauer V. (2012). Systemic inflammatory response syndrome predicts increased mortality in patients after transcatheter aortic valve implantation. *European Heart Journal*.

[16] Seo I. H., Lee Y. J. (2022). Usefulness of complete blood count (CBC) to assess cardiovascular and metabolic diseases in clinical settings: a comprehensive literature review. *Biomedicines*.

[17] Trtica Majnaric L., Guljas S., Bosnic Z., Seric V., Wittlinger T. (2021). Neutrophil-to-Lymphocyte ratio as a cardiovascular risk marker may Be less efficient in women than in men. *Biomolecules*.

[18] Lang R. M., Badano L. P., Mor-Avi V. (2015). Recommendations for cardiac chamber quantification by echocardiography in adults: an update from the American Society of Echocardiography and the European Association of Cardiovascular Imaging. *Eur Heart J Cardiovasc Imaging*.

[19] Rudski L. G., Lai W. W., Afilalo J. (2010). Guidelines for the echocardiographic assessment of the right heart in adults: a report from the American society of echocardiography. *Journal of the American Society of Echocardiography*.

[20] Zlotnick D. M., Ouellette M. L., Malenka D. J. (2013). Effect of preoperative pulmonary hypertension on outcomes in patients with severe aortic stenosis following surgical aortic valve replacement. *The American Journal of Cardiology*.

[21] Tang M., Liu X., Lin C. (2017). Meta-analysis of outcomes and evolution of pulmonary hypertension before and after transcatheter aortic valve implantation. *The American Journal of Cardiology*.

[22] Okuno T., Heg D., Lanz J. (2021). Refined staging classification of cardiac damage associated with aortic stenosis and outcomes after transcatheter aortic valve implantation. *European Heart Journal Quality of Care and Clinical Outcomes*.

[23] Ujihira K., Kohmoto T., Gimelli G. (2020). The impact of increased pulmonary arterial pressure on outcomes after transcatheter aortic valve replacement. *Catheterization and Cardiovascular Interventions: Official Journal of the Society for Cardiac Angiography & Interventions*.

[24] Liberale L., Badimon L., Montecucco F., Luscher T. F., Libby P., Camici G. G. (2022). Inflammation, aging, and cardiovascular disease: JACC review topic of the week. *Journal of the American College of Cardiology*.

[25] Liu S. F., Nambiar Veetil N., Li Q., Kucherenko M. M., Knosalla C., Kuebler W. M. (2022). Pulmonary hypertension: linking inflammation and pulmonary arterial stiffening. *Frontiers in Immunology*.

[26] Rabinovitch M., Guignabert C., Humbert M., Nicolls M. R. (2014). Inflammation and immunity in the pathogenesis of pulmonary arterial hypertension. *Circulation Research*.

[27] Al-Omary M. S., Sugito S., Boyle A. J., Sverdlov A. L., Collins N. J. (2020). Pulmonary hypertension due to left heart disease: diagnosis, pathophysiology, and therapy. *Hypertension*.

[28] Breitling S., Ravindran K., Goldenberg N. M., Kuebler W. M. (2015). The pathophysiology of pulmonary hypertension in left heart disease. *American Journal of Physiology Lung Cellular and Molecular Physiology*.

[29] Charalampopoulos A., Lewis R., Hickey P. (2018). Pathophysiology and diagnosis of pulmonary hypertension due to left heart disease. *Frontiers of Medicine*.

[30] Huston J. H., Shah S. J. (2022). Understanding the pathobiology of pulmonary hypertension due to left heart disease. *Circulation Research*.

[31] Yu X., Xue Y., Bian B. (2020). NLR-A simple indicator of inflammation for the diagnosis of left ventricular hypertrophy in patients with hypertension. *International Heart Journal*.

[32] Tamaki S., Nagai Y., Shutta R. (2023). Combination of neutrophil-to-lymphocyte and platelet-to-lymphocyte ratios as a novel predictor of cardiac death in patients with acute decompensated heart failure with preserved left ventricular ejection fraction: a multicenter study. *Journal of the American Heart Association*.

[33] Ding B., Liu P., Zhang F., Hui J., He L. (2022). Predicting values of neutrophil-to-lymphocyte ratio (NLR), high-sensitivity C-reactive protein (hs-CRP), and left atrial diameter (LAD) in patients with nonvalvular atrial fibrillation recurrence after radiofrequency ablation. *Medical Science Monitor*.

[34] Habib M., Thawabi M., Hawatmeh A. (2018). Value of neutrophil to lymphocyte ratio as a predictor of mortality in patients undergoing aortic valve replacement. *Cardiovascular Diagnosis and Therapy*.

[35] Shahim B., Redfors B., Lindman B. R. (2022). Neutrophil-to-Lymphocyte ratios in patients undergoing aortic valve replacement: the PARTNER trials and registries. *Journal of the American Heart Association*.

[36] Condado J. F., Junpaparp P., Binongo J. N. (2016). Neutrophil-lymphocyte ratio (NLR) and platelet-lymphocyte ratio (PLR) can risk stratify patients in transcatheter aortic-valve replacement (TAVR). *International Journal of Cardiology*.

